# Strategies behind the Establishment of a Developmental Cohort Study in the Tottori City

**DOI:** 10.2188/jea.JE20090191

**Published:** 2010-03-05

**Authors:** Tatsuya Koeda, Hiraku Ishida, Reiko Ishigami, Ariko Takeuchi, Shinako Terakawa, Katsutoshi Kobayashi, Ayumi Seki, Toshitaka Tamaru, JCS group

**Affiliations:** 1 Research Institute of Science and technology for society, Japan Science and Technology Agency, Tokyo, Japan; 2 Department of Regional Education, Faculty of Regional Sciences, Tottori University, Tottori, Japan; 3 Center for Education and Society, Tottori University, Tottori, Japan

**Keywords:** Japan Children’s Study, developmental cohort study, 5 years old

## Abstract

**Background:**

The Tottori study group, part of the JCS, presides over a community-based cohort study started when subjects—children living in Tottori City—were 5 years old. The social aspects of conducting a cohort study should also be made public, as this information is crucial for conducting community-based cohort studies.

**Methods:**

Documents pertaining to social aspects implemented by the Tottori study group between 2004 and 2008 were arranged chronologically. Information which is crucial for conducting community-based cohort studies were extracted and classified into several categories.

**Results:**

Five categories were extracted from the documents: research staff, supporting committee, recruitment, maintenance of motivation and disclosure. Implementation of the social aspects described in maintenance of motivation resulted in fewer subjects dropping out of the study and a re-recruitment rate of approximately 90%.

**Conclusions:**

The following factors are essential for a successful developmental cohort study of children: 1) A birth cohort study should be planned in hospitals with medical staff such as obstetricians and pediatricians; 2) An interdisciplinary group composed of medical or psychological clinicians and researchers with abundant experience in epidemiological study should be included; 3) If possible, an expert or widely known individual in the study’s target field should be included as a member of the study staff; 4) For long cohort studies, a researcher with expertise in school education should be included; 5) A support committee should be organized as an external part of the study team.

## PREFACE

The Japan Children’s Study (JCS) started in 2004.^1^ The Tottori study group, part of the JCS, presides over a community-based cohort study started when subjects—children living in Tottori City—were 5 years old. In order to conduct an effective cohort study, community residents must be provided with accurate and easy-to-understand information about the study. Various social aspects such as publicity, disclosure of information, and accountability are therefore essential. The Tottori study group implemented many of these social aspects between 2004 and 2008; as a result, fewer subjects dropped out of the study and a re-recruitment rate of approximately 90% was achieved.

The Tottori study group’s cohort study, focusing on cognition and sociability in childhood, has already produced a number of useful results.^2^^,^^3^ In the cohort study, Tottori Study group had four kinds of performances; questionnaire, medical examination and direct observation of four children during tag playing. However, in addition to academic data, the social aspects of conducting a cohort study should also be made public, as this information is crucial for conducting community-based cohort studies.

## OBJECTIVES

This report aims to clarify the social aspects essential to and useful in the establishment and maintenance of a developmental cohort study, and proposes effective plan establishment strategies.

## SUBJECTS AND METHODS

All documents pertaining to social aspects implemented by the Tottori study group between 2004 and 2008—notice and profile notes for various organizations, including Tottori City and Tottori Prefecture administrations, medical associations, and nursery associations; records of meetings with these organizations; recruitment records from municipal hospitals and kindergartens; and a JCS study plan—were studied. The documents were arranged chronologically and classified into several categories.

## RESULTS

Five categories were extracted from the documents: research staff, supporting committee, recruitment, maintenance of motivation and disclosure.

### 1. Research staff

In the JCS, the addition of four new full-time staff members—two researchers, one technical supporting staff member and one research assistant—was approved for each study group in charge of a cohort. In addition, four Tottori University research staff members were also included, making a study team of a total of eight people. Ideally, one of these four new researchers should be a pediatrician and another should be a researcher specializing in psychology. Technical supporting staff members are expected to play key roles as research coordinators. It was very difficult to recruit staff members with these qualifications in a local area such as Tottori City. In particular, we were unable to find a full-time pediatrician until 2 years after the study had begun—out of necessity, the staff pediatrician worked on a 3-day part-time employment schedule during the first 2 years.

The specialties of the Tottori University researchers included developmental psychology, clinical educational psychology in children and developmental behavioral pediatric medicine. These researchers had academic qualifications as well as close connections with people engaged in preschool education and pediatric nursing. We succeeded in employing a suitable technical research staff member who was an expert in pediatric nursing and had many years of valuable experience working in a day nursery and kindergarten in the suburbs of Tottori City. This staff member had earned the trust of the children and other employees, and the staff member’s name was widely known among the children’s parents. This individual was trusted to provide accurate advice regarding child care.

### 2. Supporting committee

When a cohort study targets diseases such as cancer or diabetes, it is relatively easy for participants and community members to understand the study’s findings and objectives. JCS, however, aims to investigate the cognitive and behavioral development and the development of sociability among children, and this goal is difficult to concretely communicate to the general public. For this reason, the full purpose of JCS was not understood completely by all of the subjects’ parents, and there were concerns that doubts or objections might emerge. In order to effectively refute any opposition to JCS, it is necessary—with the support of the organization that has supported and protected JCS—to provide a clear and easy-to-understand explanation of the purpose of the study.

“The Tottori Children’s Social Committee” was created with this goal in mind. The committee consists of an administrative division, including a welfare section and the Tottori Prefecture and Tottori City boards of education, a medical division, and a pediatric nursing division, as well as experts in maternal and child health and researchers from Tottori University. The section manager of the Tottori Welfare Health Department Maternal and Child Health Relations acts as chairperson. The committee meets twice a year and aims to make the purpose of JCS known to the citizens of Tottori Prefecture by organizing activities—such as an annual children’s forum that provides information about child care and the results of JCS—that contribute to the smooth completion of the study.

### 3. Recruitment

JCS, which was started in 2004, initially included plans for cohort studies of both infants and 5-year-olds in the Tottori study group. Babies who participated in JCS at prefectural and municipal hospitals were recruited. However, only 17% of parents provided informed consent during a recruiting period of 4 months. Other cohort groups (Osaka and Mie study groups) that included infant cohort studies succeeded in obtaining a higher rate of informed consent, as they included staff members who worked at hospitals. The difference in the recruitment success rates observed in these different cohorts suggests that the attributes of the researchers can significantly influence the outcome of recruitment activities—parents are likely to cooperate when a physician working at the hospital where their child was born requests the child’s participation in a study; however, naturally, parents are more hesitant to allow their infant to participate when the request and explanation of the study come from a researcher who is unrelated to the hospital.

After failing to recruit a suitable number of babies, the focus of the Tottori study group was shifted to 5-year-old children. Fortunately, the staff researchers from Tottori University were experts in both infant development and preschool education. Recruitment for study subjects was conducted through a kindergarten and day nursery in Tottori City. In addition, with the cooperation of Tottori City, we enclosed a recruitment handbill in medical examination notices for 3-year-old children in order to recruit future study subjects. As a result, we were able to obtain 113 subjects who were 5 years old at the start of the 2005 study and 82 who were 5 years old at the start of the 2007 study.

### 4. Motivation maintenance

In a cohort study, maintaining the participation of study subjects in line with the study objective is very important. To this end, the results of research should be returned to subjects to show them how their cooperation is contributing to the study goals. In order to accomplish this, research results from our cohort study were returned and child care information was provided at the annual children’s forum. Table 1 summarizes the number of participants in the 1st to 5th annual children’s forums.

**Table 1.** Number of annual children’s forum attendees

Forum year	Attendees
1st	100
2nd	224
3rd	274
4th	109
5th	135

New Year’s cards were sent home with study subjects each year. In response, some children sent back a New Year’s card with their photos. In 2007, when subjects started elementary school, a congratulatory greeting card was also sent. After students started elementary school, observations had to be performed during summer holidays, as there was no time for observation during the school term. We informed the principal of the subjects’ elementary school that the observations would be performed during summer holidays.

Although this cohort study was not an interventional study, several applicants who wanted counseling contacted us, and child care counseling for problems encountered during child care was sometimes carried out by staff members.

Implementation of the social aspects described above resulted in fewer subjects dropping out of the study and a re-recruitment rate of approximately 90% ranged 86.6 to 91.2%. Table 2 shows the number of subjects in the cohort study.

**Table 2.** Number of cohort study subjects

	4 mo	5 y	6 y	7 y	8 y
2004	14				
2005		113			
2006			103		
2007		82		90	
2008			71		81

### 5. Disclosure

After a local paper featured our cohort study, the study gradually became more popular in the Tottori region. Citizens who expressed interest in the study and wished to visit to the observation room were invited without exception. Specifically, 14 social education directors from the Tottori Prefectural Board of Education contacted us, wishing to see the observation room, and were invited to visit. Upon a request from the Tottori City assembly, seven members of the Nara Prefectural Assembly also made a visit. Efforts have been made to maintain the transparency of study activities.

## DISCUSSION

The plan for this cohort study specifies that subjects must be children in the general population of Tottori City. At first, it was difficult for community members to understand the purpose and significance of the cohort study—the concept of a cohort study is not widely known among the general public, and there was even less interest in developmental cohort studies in the Tottori City. The first problem to be solved is widely publicizing the goals and findings of the cohort study; otherwise, subject recruitment cannot proceed and the cohort study will prove difficult to maintain. The Tottori study group implemented a number of social aspects in order to solve these problems. These aspects are essential for developmental cohort studies with precise study plans.

In conclusion, the following factors are essential for a successful developmental cohort study of children:

1) A birth cohort study should be planned in hospitals with medical staff such as obstetricians and pediatricians;
2) An interdisciplinary group composed of medical or psychological clinicians and researchers with abundant experience in epidemiological study should be included;
3) If possible, an expert or widely known individual in the study’s target field should be included as a member of the study staff;
4) For long cohort studies, a researcher with expertise in school education should be included;
5) A support committee should be organized as an external part of the study team.
